# Aberrant T Cell Signaling and Subsets in Systemic Lupus Erythematosus

**DOI:** 10.3389/fimmu.2018.01088

**Published:** 2018-05-17

**Authors:** Takayuki Katsuyama, George C. Tsokos, Vaishali R. Moulton

**Affiliations:** Division of Rheumatology, Department of Medicine, Beth Israel Deaconess Medical Center, Harvard Medical School, Boston, MA, United States

**Keywords:** SLE, autoimmunity, signaling, T cells, Autoimmune disease

## Abstract

Systemic lupus erythematosus (SLE) is a chronic multi-organ debilitating autoimmune disease, which mainly afflicts women in the reproductive years. A complex interaction of genetics, environmental factors and hormones result in the breakdown of immune tolerance to “self” leading to damage and destruction of multiple organs, such as the skin, joints, kidneys, heart and brain. Both innate and adaptive immune systems are critically involved in the misguided immune response against self-antigens. Dendritic cells, neutrophils, and innate lymphoid cells are important in initiating antigen presentation and propagating inflammation at lymphoid and peripheral tissue sites. Autoantibodies produced by B lymphocytes and immune complex deposition in vital organs contribute to tissue damage. T lymphocytes are increasingly being recognized as key contributors to disease pathogenesis. CD4 T follicular helper cells enable autoantibody production, inflammatory Th17 subsets promote inflammation, while defects in regulatory T cells lead to unchecked immune responses. A better understanding of the molecular defects including signaling events and gene regulation underlying the dysfunctional T cells in SLE is necessary to pave the path for better management, therapy, and perhaps prevention of this complex disease. In this review, we focus on the aberrations in T cell signaling in SLE and highlight therapeutic advances in this field.

## Introduction

**Figure 1:**
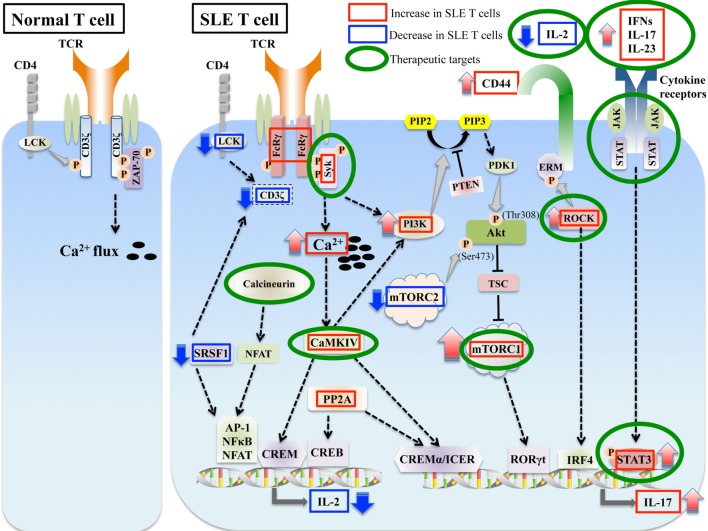
Aberrant signaling in systemic lupus erythematosus (SLE) T cells. SLE T cells are characterized by multiple aberrant signaling pathways, such as decreased CD3ζ, activated PI3K-Akt-mTORC1 pathway, Rho associated protein kinase (ROCK), calcium/calmodulin kinase IV (CaMKIV), and protein phosphatase 2A (PP2A). These are associated with abnormalities in T cell differentiation and production of proinflammatory cytokines such as IL-17 and decreased production of vital cytokines such as IL-2. Molecules aberrantly increased or decreased in SLE are indicated in red and blue boxes, respectively, and molecules that are potential therapeutic targets are in green circles.

Systemic lupus erythematosus (SLE) is a complex systemic autoimmune disease involving multiple organs leading to tissue damage and diverse clinical manifestations. Although the etiology of SLE is still unclear, a number of recent studies have advanced our understanding of disease pathogenesis. Clinical heterogeneity of SLE suggests that there are number of players in the immune system that contribute to the pathogenesis of SLE. B cells obviously are important in autoimmune diseases through the production of antibodies by plasma cells and presenting antigens to T cells. However, there is an increasing recognition and validation of the critical role of T cells in SLE pathogenesis (1–5). Historically, the T helper (Th)1/Th2 balance was considered to be important in the pathogenesis of SLE (6, 7). However, recent understanding of the detailed mechanisms of T cell differentiation and subsets have elucidated the more important and complicated role of T cells in the pathogenesis of this autoimmune disease. Many studies have shown abnormal cytokine production and aberrant cell signaling in SLE T cells, which dictate not only the abnormalities in T cell differentiation but also the excessive activation of B cells. It is expected that these abnormal signaling molecules can serve as therapeutic targets for the treatment of patients with SLE. In this review, we focus on signaling molecules and pathways in T cells from SLE patients and lupus-prone mice, and highlight those that can be exploited therapeutically (Figure 1).

## T Cell Receptor (TCR)

The TCR is a heterodimer, consisting of the TCRα and TCRβ chains in most cells, which recognizes antigenic peptides presented by the major histocompatibility complex (MHC) on antigen presenting cells. The TCR is assembled with a complex of CD3 proteins (CD3δ, ε, γ, and ζ). CD3δ, ε, and γ are members of the immunoglobulin superfamily and genetically related to each other, whereas CD3ζ subunit is genetically and structurally unrelated to the other CD3 subunits (8–10). CD3ζ contains three immunoreceptor tyrosine-based activation motif (ITAM) domains, and the phosphorylation of ITAM residues is a key step in the complex process of TCR signaling. Following TCR recognition and engagement of the MHC—antigen complex, the Src kinase lymphocyte-specific protein tyrosine kinase (Lck) phosphorylates ITAMs of CD3ζ. Phosphorylated CD3 ITAMs recruit the spleen tyrosine kinase (Syk) family kinase ζ-associated protein kinase 70 (ZAP-70) *via* Src-homology 2 domain, and Lck phosphorylates the bound ZAP-70, resulting in the activation of ZAP-70 (11). Activated ZAP-70 phosphorylates tyrosine residues on the adaptor proteins linker for activation of T cells (LAT) and SLP-76, which bind and activate phospholipase Cγ (PLC-γ). Activated PLC-γ hydrolyzes phosphatidylinositol-4,5-bisphosphate (PIP2) to produce inositol 1,4,5-trisphosphate and diacylglycerol, resulting in the calcium flux and the activation of protein kinase C (PKC) and Ras-mitogen-activated protein kinase pathway through the recruitment of Ras guanine releasing protein 1 (12, 13).

The expression levels of CD3ζ chain are significantly decreased in T cells from SLE patients (14–16), and this defect coupled with a rewiring of the TCR complex, contributes to the aberrant signaling phenotype of SLE T cells. In association with the reduced levels of CD3ζ protein in SLE T cells, the TCR–CD3 complex bears a substitution by the homologous Fc receptor common gamma subunit chain (FcRγ), which is not normally expressed in resting T cells. Although FcRγ was identified as a component of the high affinity IgE receptor (FcεRI), it is now recognized as a common subunit of other Fc receptors (17, 18). FcRγ is upregulated upon activation in effector T cells (19–22). CD3ζ and FcRγ are structurally and functionally homologous (23). FcRγ recruits Syk instead of ZAP-70, which is normally recruited by CD3ζ. FcRγ–Syk interaction is significantly stronger than CD3ζ–ZAP-70 interaction, resulting in the higher calcium influx into T cells (14, 21). Reconstitution of CD3ζ in SLE T cells restores the aberrant signaling and calcium flux (24). Interestingly, CD3ζ-deficient mice spontaneously develop multi-organ tissue inflammation (25). Therefore, the reduced expression levels of CD3ζ are important in the aberrant T cell signaling phenotype, and understanding the mechanisms leading to its downregulation would help target those factors to correct the T cell signaling defect. A number of mechanisms for the downregulation of CD3ζ mRNA and protein in T cells from SLE patients have been elucidated. In addition to abnormalities in transcription (14, 26), aberrant alternative splicing (27–29) and stability (30, 31) of CD3ζ mRNA contribute to the decreased expression levels of CD3ζ protein in T cells from SLE patients. Serine/arginine-rich splicing factor 1 (SRSF1), also known as splicing factor 2/alternative splicing factor controls the alternative splicing (32) and contributes to the transcriptional activation (33) of CD3ζ, to promote normal expression of CD3ζ protein. Decreased SRSF1 expression in T cells from SLE patients correlates with worse SLE disease activity (34), and with reduced CD3ζ levels. Recently, it was reported that hypermethylation marks are present within the CD3ζ gene promoter in SLE patients (35). These findings suggest that CD3ζ hypermethylation may contribute to the downregulation of CD3ζ in T cells from SLE patients.

The serine/threonine protein phosphatase 2A (PP2A) is a ubiquitous serine-threonine phosphatase and composed of three distinct subunits; the scaffold A subunit (PP2A_A_), the regulatory B subunit (PP2A_B_), and the catalytic C subunit (PP2A_C_) (36). PP2A controls the expression of CD3ζ and FcRγ at the transcription level through the dephosphorylation of Elf-1 (37). In T cells from SLE patients, increased PP2Ac activity results in aberrant TCR signaling leading to abnormal T cell function.

## Proximal TCR Signaling

TCR-CD3 engagement with antigens induces the phosphorylation of ITAM residues by Lck, a member of the Src kinase family. The expression levels of Lck are decreased in T cells from SLE patients (38–41). A potential mechanism for the reduced Lck expression is its degradation due to increased ubiquitination. Lipid rafts, microdomains in the plasma membrane enriched in cholesterol, sphingomyelin, and glycosphingolipids, play important role in TCR signaling (42, 43). Lck localizes to lipid rafts, and accumulation of lipid rafts induces the increased phosphorylation and signal transduction (44, 45). Freshly isolated SLE T cells express higher levels of ganglioside M1 and cholesterol, a component of raft domain, and aggregated lipid rafts (46–48). Atorvastatin, which reduces cholesterol synthesis, restores Lck expression and lipid raft-associated aberrant signaling *in vitro* in T cells from patients with SLE (49). Atorvastatin also reduces the production of IL-10 and IL-6 by activated T cells (49).

Phosphorylation of ITAM residues of the TCR-CD3 complex molecules following antigen recognition by the TCR leads to the recruitment and activation of downstream signaling molecules such as adaptor proteins and enzymes. As described above, phosphorylated ITAMs of CD3ζ serve as a recruitment site for tyrosine kinase ZAP-70, a member of the Syk kinase family (50). It is unclear whether ZAP-70 expression levels T cells from SLE patients are comparable to those in T cells from healthy individuals (51) or decreased (52).

In addition to its role in T cell signaling, Syk is also an important molecule downstream of the B cell receptor. Expression levels of Syk and phospho (p)-Syk in B cells from active SLE patients are increased compared with controls (53). Therefore, Syk inhibitors are promising therapeutics. Fostamatinib, also known as R788 is a small molecule pro-drug of the biologically active R406 (54, 55), which selectively inhibits Syk. Inhibition of Syk by fostamatinib prevents disease development including skin and renal involvement in MRL/lpr and BAK/BAX lupus-prone mice, and the discontinuation of the treatment results in extended suppression of renal disease for at least 4 weeks (56). The administration of fostamatinib after the development of disease also improves kidney damage in New Zealand black/white (NZB/NZW) lupus-prone mice (57). Further studies are required to assess the efficacy of Syk inhibitors in patients with SLE.

Enhanced early T cell signaling events and heightened calcium responses lead to increased activation of calcineurin. Calcineurin dephosphorylate inactive cytoplasmic nuclear factor of activated T cells (NFAT) and dephosphorylated NFAT translocates to the nucleus. Increased recruitment of NFATc2 is observed in the nuclei of activated T cells from SLE patients after CD3 stimulation compared with those from controls, and it binds and activates the promoters of CD154 (CD40L) and IL2 genes (58). CD40-CD40L signaling is also important for the differentiation of Th17 cells (59). Expression of NFATc1 is elevated in lupus-prone MRL/lpr mice (60). Dipyridamole, an inhibitor of the calcineurin-NFAT pathway, reduces CD154 expression and improves nephritis in MRL/lpr mice (60). Calcineurin inhibitors cyclosporine and tacrolimus are widely used for the treatment of SLE. They are known to be effective in the treatment of lupus nephritis as both remission induction and maintenance therapy (61).

## CD44-ROCK-ERM Axis

CD44 is a cell surface glycoprotein involved in T cell activation, adhesion, and migration (62). Recent genome wide association studies (GWAS) have identified CD44 as a gene associated with SLE on meta-analysis of two SLE GWAS datasets by OASIS, a novel linkage disequilibrium clustering method (63). It was also reported that the expression levels of CD44 are increased in T cells from SLE patients (48, 64). The CD44 gene includes 10 variable (v) exons and there are numerous splice variants of CD44. CD44v3 and CD44v6 are expressed on T cells following activation (65, 66). The expression levels of CD44v3 and CD44v6 are increased and correlate with disease activity in patients with SLE (64).

The ezrin/radixin/moesin (ERM) proteins are important in linking plasma membrane proteins with actin filaments, and the interaction between ERM proteins and the intracellular domain of CD44 is associated with cell adhesion and migration function (67). T cells predominantly express ezrin and moesin (68). Moesin-deficient mice, which exhibit significantly lower levels of pERM (69), develop systemic autoimmune phenotype including glomerulonephritis (70), and exhibit reduced CD8^+^CD44^+^CD122^+^Ly49^+^ regulatory T (Treg) cells and defects in the signal transducer and activator of transcription (STAT) 5 activation by IL-15, which is known to regulate the development of CD8 Treg cells. The levels of ERM phosphorylation are increased in SLE T lymphocytes, and forced expression of constitutively active ezrin enhances the adhesion and migration in normal T cells, suggesting that phosphorylated ERM is responsible for increased adhesion and migration of SLE T cells (48).

Rho associated protein kinase (ROCK) is a serine/threonine kinase that phosphorylates ERM. The ROCKs play important roles in migration, activation, and differentiation of T cells (71). ROCKs are a family of two serine-threonine kinases, ROCK1 and ROCK2, which exhibit a high degree of identity in their kinase domains (72). ROCKs regulate the activity of cytoskeletal components including ERM and cell migration. ROCK activity is important for chemokine-mediated polarization and transendothelial migration of T cells (73). Recently, it was reported that ROCK also regulates the interstitial T cell migration (74). In addition to its role in T cell migration, ROCK2 plays an important role in the differentiation of Th17 cells by activation of interferon regulatory factor 4 (IRF4) and controls the production of IL-17 and IL-21 (75, 76). ROCK2 signaling is also required for the induction of T follicular helper cells (Tfh cells) (77). Peripheral blood mononuclear cells (PBMC) from patients with SLE express significantly higher levels of ROCK activity as compared with healthy controls (78, 79).

In accordance with these results, ROCK inhibitors are candidates to be used for the treatment of patients with SLE. KD025 is a selective ROCK2 inhibitor (80), whereas Y-27632 (81), and Simvastatin are broad non-isoform selective ROCK inhibitors (82). Oral administration of KD025 to healthy subjects in a randomized phase I clinical trial, decreased the production of IL-17 and IL-21 from human T cells (76). KD025 also reduced the number of Tfh cells and autoantibody production in MRL/lpr mice (77). Y-27632 decreased serum levels of IL-6, IL-1β, and TNF-α and increased serum levels of IL-10 and Treg cell proportions in spleen cells from MRL/lpr mice, whereas the improvement of clinical manifestations was not shown in the paper (83). Rozo et al. demonstrated that each Y-27632, KD025 or simvastatin inhibits the increased ROCK activity in Th17 cells from SLE patients. These agents also decreased the production of IL-17 and IL-21 from SLE T cells or Th17 cells (79).

Fasudil, a pan ROCK inhibitor, has been approved for clinical use in Japan and China for the improvement of cerebral vasospasm after surgery for subarachnoid hemorrhage (71, 84). Fasudil decreases the production of IL-17 and IL-21 and improve disease including production of autoantibody and proteinuria in MRL/lpr mice (75), and NZB/W F1 mice (85). These results indicate that ROCK signaling is a promising therapeutic target for patients with SLE.

## Phosphoinositide-3 Kinases (PI3Ks) and Phosphatase and Tensin Homolog Deleted on Chromosome 10 (PTEN)

Class I PI3Ks, family members of lipid kinases, are classified as class IA and IB by activation mode. Class IA PI3Ks are activated by receptor tyrosine kinases including the TCR and costimulators, whereas Class IB PI3Ks are activated by G protein-coupled receptors such as chemokine receptors (86–88). Class I PI3Ks are composed of catalytic subunits p110 and regulatory subunits p85 or p87. There are three catalytic isoforms of Class IA PI3Ks (p110α, p110β, and p110δ), whereas only p110γ is a PI3K Class IB catalytic subunit. Compared with the ubiquitous expression of p110α and p110β, p110δ and p110γ are selectively expressed in lymphocytes (89). Class I PI3Ks phosphorylate PIP2 to form phosphatidylinositol-3,4,5-triphosphate (PIP3). Both Class IA and IB PI3Ks are expressed in leukocytes and play important roles in homeostasis, differentiation and function of T cells (88, 90, 91). PIP3 recruits phosphoinositide-dependent kinase 1 and activates Akt.

Phosphoinositide-3 kinase plays an important role in T cell differentiation (92). Transgenic mice expressing an active form of PI3K in T cells, p65^PI3K^ Tg mice, develop lupus-like autoimmune phenotypes including kidney disease (93). Cleaved CD95 (Fas) ligand (CD95L/FasL) is increased in serum from patients with SLE and promotes cell migration through a c-yes/Ca^2+^/PI3K signal (94). Class I PI3K signaling is activated in lymphocytes of MRL/lpr mice, and treatment with AS605240, a PI3Kγ selective inhibitor, reduces the severity of glomerulonephritis and prolongs lifespan in these lupus-prone mice, indicating an important role of PI3K signaling in SLE pathogenesis (95). Activation of PI3Kp110δ is enhanced in T cells from SLE patients, and the activation of PI3K pathway is associated with the defect of activation-induced cell death (AICD) in SLE T cells (96). PI3Kδ inhibition by GS-9289, a selective inhibitor of p110δ subunit, prolongs life span and reduces kidney damage in MRL/lpr mice (97), and general PI3K inhibition by Ly294002 rescues the AICD defect in T cells from SLE patients (96), suggesting that PI3K inhibitors may be potentially important drugs to treat patients with SLE.

Phosphatase and tensin homolog deleted on chromosome 10 dephosphorylates PIP3 and regulates the PI3K/Akt pathway (98). PTEN was originally reported as a tumor suppressor gene in 1997 (99–101), and T-cell-specific PTEN deficient mice exhibit increases in thymic cells and develop T-cell-derived lymphomas (102, 103). Treg-specific PTEN deficient mice show autoimmune phenotypes by loss of Treg function and stability (104, 105). On the other hand, the role of PTEN in Th17 cell differentiation is controversial. Overexpression of PTEN inhibits STAT3 activation and Th17 differentiation, and ameliorates the development of collagen-induced arthritis (106). By contrast, Th17-specific PTEN deficient mice exhibit impaired *in vitro* Th17 cell differentiation and mitigated symptoms of experimental autoimmune encephalomyelitis (107). PTEN deficiency increases the production of IL-2 and phosphorylation of STAT5, but reduces STAT3 phosphorylation, suggesting that further studies are required to determine the exact role of PTEN in T cell differentiation and the activation of STAT signals.

There is limited evidence demonstrating how PTEN is associated with the pathogenesis of SLE. Overexpression of miR-148a-3p, which is increased in the glomeruli of patients with lupus nephritis, induces mesangial cell proliferation in glomeruli and reduces the expression level of PTEN (108). Also, SLE B cells exhibit decreased expression levels of PTEN, which inversely correlates with disease activity (109), whereas there is no clear evidence available to elucidate the role of PTEN in SLE T cells.

## Mechanistic Target of Rapamycin (mTOR) Pathway

Mechanistic target of rapamycin, a ubiquitous serine-threonine kinase, integrates environmental cues from a variety of pathways to regulate various cellular processes including cellular survival, proliferation and differentiation, and cellular metabolism (110, 111). mTOR is a component of two distinct complexes, mTOR complex (C)1 and mTORC2. The components of mTORC1 are mTOR, regulatory protein associated with mTOR (Raptor), mammalian lethal with Sec13 protein 8 (mLST8) and inhibitory subunits proline-rich Akt substrate of 40 kDa and DEP domain containing mTOR-interacting protein (DEPTOR). mTORC2 also contains mTOR, mLST8, DEPTOR, whereas it is composed of rapamycin insensitive companion of mTOR (Rictor), instead of Raptor, and inhibitory subunits mammalian stress-activated protein kinase interacting protein 1 and Protor (protein observed with Rictor) 1/2 (112). mTORC1 phosphorylates two key effectors for protein synthesis; p70S6 kinase 1 (S6K1) and EIF4E binding protein, whereas mTORC2 phosphorylates serum- and glucocorticoid-induced kinase 1, Akt (Ser473), and PKC.

Mechanistic target of rapamycin plays an important role in cellular metabolism (113). mTORC1 increases the translation of the transcription factor hypoxia-inducible factor 1α, which induces glycolytic genes (114). Glycolysis is elevated in CD4+ T cells from lupus-prone (B6.*Sle1.Sle2.Sle3* mice and B6.*lpr* mice) and SLE patients (115, 116). mTORC1 also regulates both general autophagy and mitophagy, which are important in maintaining mitochondrial function (117). T cells from SLE patients exhibit increased mitochondrial mass and mitochondria dysfunction, characterized by elevated mitochondrial transmembrane potential (118, 119). Increased mitochondrial metabolism in SLE T cells can contribute to aberrant T cell function (111). Along these lines, normalization of CD4^+^ T cell metabolism by mitochondrial metabolism inhibitor metformin and the glucose metabolism inhibitor 2-Deoxy-d-glucose reduced IFNγ production from CD4^+^ T cells *in vitro* and suppressed autoimmunity and nephritis in B6.*Sle1.Sle2.Sle3* mice and NZB/W F1 mice (115).

Recent studies have proven the important role of mTOR in the polarization of T cells. Th1 and Th17 differentiation is selectively regulated by mTORC1 signaling (120), and the inhibition of mTOR *in vivo* reduces the proportion of Th1 cells and Th17 cells in the lamina propria and mesenteric lymph nodes (121). It is also reported that both mTORC1 and mTORC2 are essential for Tfh cell differentiation and germinal cell reaction under steady state and after antigen immunization and viral infection (122).

The role of mTOR in Treg differentiation is complicated. mTORC1 signaling is constitutively active in Treg cells and its disruption in Treg cells leads to profound loss of Treg suppressive activity, although mTORC1 does not directly impact the expression of Foxp3 (123). On the other hand, both mTORC1 and mTORC2 suppress induced-Treg generation *in vitro* (120, 124). PP2A activation induces the inhibition of the mTORC1 pathway but has no effect on the mTORC2 pathway, and Treg cell-specific ablation of the PP2A results in a severe systemic autoimmune disorder through Treg dysfunction (125).

Recently, it has been recognized that activation of the mTOR pathway plays an important role in the pathogenesis of autoimmune diseases including SLE (119). mTORC1 activity is increased in the livers of MRL/lpr mice (126). In SLE T cells, mTORC1 activity is increased while mTORC2 is reduced compared with T cells from healthy donors (127). Tuberous sclerosis complex (TSC), an autosomal dominant disorder, affects multiple organ systems resulting from mutations in either of TSC 1 or TSC2 genes, which negatively regulate mTORC1 activation (128). Singh et al. reported a fatal lupus patient complicated with TSC, suggesting that mTORC1 activation led to the development of unusually severe SLE (129). Therefore, mTOR has become a therapeutic target in SLE. Rapamycin, the best-known inhibitor of mTOR, has been approved by the FDA to preserve renal allografts (111). Recent studies have uncovered the effect of rapamycin on SLE T cells *in vitro*. Increased IL-17 expression in CD4+ T cells from SLE patients is suppressed and Treg cells are expanded by rapamycin (127, 130). SLE Treg cells exhibit increased mTORC1 and mTORC2, and IL21 stimulates mTORC1 and mTORC2 and blocks the differentiation of Treg cells (131). Rapamycin reduces both the activation of STAT3 and the number of IL-17 producing cells in patients with SLE (132), and decreases the severity of lupus nephritis and prolongs survival in MRL/lpr mice (133). There are reports of studies with small numbers of patients with SLE showing the efficacy of oral administration of rapamycin (22, 134). Importantly, the deficiency of the CD3ζ chain and upregulation of FcεRIγ chain and Syk in T cells from SLE patients *in vitro* are reversed by rapamycin treatment (22).

*N*-acetylcysteine (NAC), a precursor of glutathione, is another inhibitor of mTOR. A randomized double blind placebo-controlled study to assess the efficacy and the safety of NAC in SLE patients (135), demonstrated that 2.4 and 4.8 g daily NAC reduced disease activity and mTOR activity, reversed the expansion of CD3+ CD4-CD8- double negative (DN) T cells, and stimulated Foxp3 expression in CD4^+^CD25^+^ T cells. There are other reports showing the efficacy of NAC in SLE patients with lupus nephritis (136, 137).

Overall, mTOR inhibitors are accepted as a novel class of drugs that can target both cellular signaling and metabolism. To establish the efficacy of mTOR inhibitors in SLE patients and identify patients who respond to treatment, further studies with larger number of patients are necessary. Recently, results of a large prospective open-label, phase 1/2 trial of rapamycin (Sirolimus) in patients with active SLE were reported (138). During the course of 12 months of treatment, disease activity scores reduced in 16 (55%) of 29 patients treated with Sirolimus. Sirolimus treatment expanded Tregs and CD8^+^ memory T cell populations and inhibited IL-4 and IL-17 production by CD4^+^ and DN T cells. Although this study is a single-arm study and placebo-controlled clinical trials with increased number of patients are required, the trial suggests that mTOR blockade may be a promising therapeutic target in the treatment of SLE.

## Cytokine Signaling

Cytokines play critical roles in the proliferation, activation, differentiation, and function of T cells. The Janus kinase (JAK)–STAT signaling pathway following cytokine-receptor activation is one of the most important pathways used by multiple cytokines. In humans, seven STAT family members have been identified (STAT1, STAT2, STAT3, STAT4, STAT5A, STAT5B, and STAT6) (139). Different cytokines can activate specific STATs, and STATs regulate transcription of various genes including master regulators of differentiated T cell subsets. STAT1/STAT4 activate Tbet, the transcription factor which drives Th1 cell differentiation, STAT6 induces GATA3 in Th2 differentiation, STAT3 activates RORγt which activates IL-17 and Th17 differentiation, STAT3 induces Bcl6 transcription factor of Tfh cells, and STAT5 activates Foxp3 which drives Treg differentiation (140). STAT proteins are, therefore, essential for the establishment of lineage-specific enhancer landscapes of differentiating T cells (141). A number of studies have shown that STAT signaling plays a critical role in autoimmune diseases including SLE (142).

### STAT1 and Interferons

A number of studies have revealed that IFNs play important roles in lupus pathogenesis (143, 144). The phosphorylation of STAT1, which is activated by all types of IFNs, is increased in MRL/lpr mice (145, 146). Consistent with these results, it was observed that the expression levels of STAT1 are increased in leukocytes from SLE patients (147–149). The expression levels of miR-145, a suppressor of STAT1, are decreased in T cells from SLE patients, and increased levels of STAT1 in human SLE T cells are associated with lupus nephritis (150). Recently, it was reported that the levels of STAT1 protein were increased in CD4 T cells from SLE patients and positively correlated with disease activity (151), and high STAT1 phosphorylation responses were observed in activated Tregs, which were decreased in peripheral blood from SLE patients. These results suggest that STAT1 can be a therapeutic target in SLE. However, the involvement of STAT1 in SLE is complex because STAT1 deficient lupus-prone mice exhibit interstitial kidney inflammation associated with Th17 cells, by shunting to STAT3/4 activation (152).

### IL-23—STAT3—IL-17 Axis

Th17 cells produce the IL-17 cytokines IL-17A and IL-17F. Increased numbers of Th17 cells and increased levels of IL-17 have been found in patients with SLE and in lupus-prone mice (153–155). IL-17-producing cells have been found in kidney biopsies of patients with lupus nephritis (156) and in kidneys and spleen of MRL/lpr lupus-prone mice (157), and levels of IL-17 correlate with SLE disease activity (153). DN T cells are a key source of IL-17 in MRL/lpr mice (156, 157), and more importantly they are present in the kidney tissue of patients with lupus nephritis (156).

Recent studies have uncovered aberrant mechanisms associated with Th17 differentiation and IL-17 production in SLE T cells. IL-23, a member of the IL-12 family, is important for the maintenance of Th17 cells. Serum levels of IL-23 are increased in patients with SLE with high disease activity (158). IL-23 induces the activation of STAT3 (159–161). STAT3 directly binds the promoters of *IL-17A* and *IL-17F* (162), and T cell-specific deletion of STAT3 reduces IL-17 expression and impairs RORγt expression (163). STAT3 is upregulated and activated in both lupus-prone mice (164, 165) and T cells from patients with SLE (166, 167).

In addition to its role in Th17 differentiation, STAT3 is also important for the development of follicular helper T cells (Tfh cells), which induce the differentiation of germinal center B cells into memory and antibody-secreting cells (168). Tfh cells are expanded in both patients with SLE and lupus-prone mice (169). STAT3 also plays a role in the production of other cytokines including IL-10, which promotes B-cell proliferation and antibody production, and is elevated in the serum and kidneys of patients with SLE (167, 170–172). STAT3 was shown to promote IL-10 expression through trans-activation and chromatin remodeling of the *IL-10* locus in T cells from patients with SLE (167).

Therefore, STAT3 inhibitors could be promising therapeutic candidates to treat patients with SLE. Indeed, administration of a STAT3 inhibitor to MRL/lpr mice delays the onset of lupus nephritis in Ref. (173).

Janus kinase inhibitors are also promising therapeutic agents. JAK2 inhibitor AG490 suppressed the production of anti-histone/dsDNA antibodies in short-term culture (174). Tofacitinib is an oral JAK inhibitor, which inhibits JAK1, JAK3 (to a less extent), and JAK2, and has been approved for the treatment of rheumatoid arthritis. Tofacitinib improves disease activity of lupus-prone mice including nephritis, skin inflammation, and autoantibody production (175, 176). Baricitinib, another JAK inhibitor, is also under investigation for the treatment of SLE (177).

There are some reports indicating that IL-23 contributes to organ inflammation independent of its contribution to Th17 differentiation. IL-23 is important in the development of T cell-dependent colitis (178), yet IL-23-dependent colitis does not require IL-17 secretion by T cells, because CD4^+^ CD45RB^hi^ T cells cannot induce colitis in *Il23a^−/−^* Rag1^−/−^ recipients even though intestinal IL-17 is unaffected by the absence of IL-23 (179). Furthermore, although IL-23 is not essential for the expression of Foxp3, IL-23 can have an indirect effect on Treg cell generation. IL-23 receptor deficiency in lupus-prone mice results in decreased production of anti-dsDNA antibodies and proliferation of DN T cells (180, 181). Interestingly, IL-23 not only promotes IL-17 production but also decreases the production of IL-2 by impairing the *Il2* gene enhancer NFκBp65 in mice (181). Also, IL-23 stimulation expands DN T cells from SLE patients *in vitro* (182). A phase IIa trial of Ustekinumab, targeting the p40 subunit common to IL-12 and IL-23, is underway in patients with SLE ((183). Inhibition of IL-23 signaling by an anti-IL-23p19 antibody ameliorates nephritis in MRL/lpr mice (184). Tildrakizumab (MK-3222), a monoclonal antibody targeting the p19 subunit, is under investigation for treatment of moderate-to-severe chronic plaque psoriasis (185, 186). Another monoclonal antibody targeting the p19 subunit, MEDI2070 (also known as AMG 139), improved clinical activity of Crohn’s disease in a phase IIa trial (187), although no data are available yet in patients with SLE.

There are other factors related to Th17 differentiation in SLE T cells. PP2A controls various signaling pathways, and CD4 T cells from transgenic mice that overexpress the catalytic subunit of PP2A in T cells produce increased amounts of IL-17 (188). The cAMP response element modulator (CREM) family of transcription factors also plays an important role in the differentiation of Th17 cells and IL-17 production. The suppressor isoform CREMα, which is increased in SLE T cells, reduces CpG-DNA methylation of the *IL-17A* locus, and controls IL-17A expression (189). Inducible cAMP early repressor (ICER), a transcriptional repressor isoform of CREM, is important for Th17 cell differentiation. ICER binds to the *IL-17A* promoter and enhances accumulation of the canonical IL-17 transcription factor RORγt (190). Calcium/calmodulin kinase IV (CaMKIV) is activated in T cells from SLE patients and MRL/lpr mice (191–193), and promotes the differentiation of Th17 cells and IL-17 production by activating the Akt/mTOR pathway (130). In MRL/lpr mice, genetic deletion of CaMKIV prolongs survival, and CaMKIV inhibitor KN-93 leads the suppression of nephritis and skin disease (192, 193). Moreover, as described above, ROCK is also associated with Th17 differentiation and production of IL-17 through the activation of IRF4 (75, 76).

Secukinumab and Ixekizumab are monoclonal antibodies targeting IL-17A while Brodalumab targets the IL-17A receptor, thus inhibiting the IL-17 signaling pathway. Although the evidence is clear for the efficacy and safety of these agents in the treatment of psoriasis and ankylosing spondylitis (194), there are no data showing efficacy of inhibition of IL-17 in SLE patients so far. Despite the overwhelming evidence that IL-17 contributes to lupus pathology, IL-17A deficiency in lupus-prone MRL/lpr mice or IL-17A neutralization in NZB/NZW mice did not affect the course of nephritis (195). Further work is needed to dissect the role of this signaling pathway in lupus pathogenesis in order to target it effectively.

### STAT5 and IL-2

IL-2 is a key cytokine important in the proliferation, activation, and differentiation of T cells (196). Importantly, IL-2 plays a vital role in the homeostasis of Treg cells. Mice and humans deficient in IL-2, IL-2Rα (CD25), or IL-2Rβ (CD122) develop systemic autoimmunity due to impaired Treg cells (197–203). Also, IL-2 negatively regulates IL-17 production *in vivo* and *in vitro* (204, 205). In addition, IL-2 inhibits the differentiation of Tfh cells through the activation of Akt-mTORC1 signaling, and instead promotes the differentiation of Th1 cells (206). IL-2 also plays a critical role in the induction of AICD, a key process responsible for the deletion of autoreactive cells (207, 208).

It has been known for a long time that the insufficient production of IL-2 from T cells is one of the most important characteristic features of both SLE patients and lupus-prone mice (209–211). The molecular mechanisms of the decreased IL-2 production from SLE T cells have not completely been elucidated, whereas a number of studies have identified several mechanisms. Various transcription factors binding to the IL-2 promoter affect the expression of IL-2. NF-κB and activator protein 1 (c-fos/c-jun heterodimer) are downregulated in T cells from SLE patients, and linked to decreased IL-2 transcription (212–214). PP2A, a ubiquitous phosphatase, is increased in SLE T cells. PP2A dephosphorylates cyclic AMP-responsive element-binding protein 1, which can directly bind to the *IL-2* promoter and reduce IL-2 production (215). CaMKIV plays a role in the shortage of IL-2 in SLE T cells as well. CaMKIV is increased in SLE T cells, and phosphorylates CREM to suppress IL-2 transcription (191). As described above, it was recently reported that PTEN deficiency increases the production of IL-2 and phosphorylation of STAT5 (107), suggesting a novel mechanism of the IL-2 deficiency in SLE T cells, whereas the role of PTEN in SLE T cells remains unclear. SRSF1 is a multifunctional protein, which contributes to the transcriptional activation of IL-2. SRSF1 levels are decreased in T cells from SLE patients, and overexpression of SRSF1 into SLE T cells, rescues IL-2 production (34). It was demonstrated that increased expression of miR-200a-3p is associated with the decreased production of IL-2 through zinc finger E-box binding homeobox–C-terminal binding protein 2 in MRL/lpr mice (216).

Although the molecules that contribute to the decreased production of IL-2 can serve as therapeutic targets for the treatment of patients with SLE, strategies to restore IL-2 levels have been exploited. Recently, the safety and efficacy of low-dose IL-2 therapy for patients with graft-versus-host disease (217, 218), type 1 diabetes (219), and cryoglobulinemia associated with HCV infection have been reported (220). There are uncontrolled reports indicating the efficacy of low-dose IL-2 therapy in patients with SLE (221–223). Treatment of MRL/lpr lupus-prone mice with an IL-2-expressing recombinant adeno-associated virus resulted in reduced pathology, decreased DN cell numbers and increased Treg cell numbers (224). Subcutaneous injection of low-dose IL-2 on five consecutive days in a small number of patients with SLE, achieved decreases in SLE Disease Activity Index (SLEDAI) and increased peripheral Treg cells (221, 225). An uncontrolled study of 37 consenting patients with SLE claims that subcutaneous administration of recombinant IL-2 every other day for 2 weeks decreased SLEDAI, Th17, Tfh, and DN T cells, and increased Treg cell numbers (222). Further studies are required to overcome the challenges of maintaining IL-2 levels due to a very short half-life of the cytokine. It is important to note that not only the production of IL-2 by T cells from patients with SLE impaired, but also the response to exogenous IL-2 is impaired in CD4 T cells compared with healthy controls (226). These results suggest that we should also consider strategies to restore IL-2 sensitivity of T cells during low-dose IL-2 therapy. Indeed, the engagement of SLAMF3 in T cells from normal subjects and patients with SLE increased their IL-2-initiated signaling strength (227).

### Transforming Growth Factor-β (TGF-β) Signaling

Transforming growth factor-β has three different isoforms (TGF-β1, 2, and 3), and regulates cell growth and differentiation. TGF-β signaling is essential for the differentiation of Treg cells. TGF-β signaling induces the expression of Foxp3 (228), and T cell-specific loss of TGF-β results in the defect in the differentiation of thymic Treg cells in mice (229). In addition, TGF-β also acts as a direct regulator against autoreactive T cells in part through the regulation of GM-CSF production (230, 231). Moreover, TGF-β also contributes to the differentiation of Th17 cells (232), whereas Th17 cells also can be generated without TGF-β signaling but with IL-6, IL-1β, and IL-23 (233).

The role of TGF-β in SLE patients remains unclear. It was reported that serum levels of TGF-β are decreased in active SLE patients (234, 235). On the other hand, some reports demonstrated that TGF-β1 production is increased from SLE PBMC (236). Impaired response of peripheral blood cells to TGF-β1 in patients with active SLE has been reported (237). CD4^+^CD25^-^Lag3^+^ Treg cells expressing early growth response gene (Egr)2 and Egr3 exhibit immune suppressive capacity by secreting TGF-β3, and mice with T cell-specific deletion of Egr2/3 mice develop lupus-like disease (238, 239). Further studies are required to uncover the role of TGF-β in the pathogenesis of lupus.

## Conclusion

A great effort has been made to delineate specific abnormalities in immune cells from SLE patients, and a dramatic expansion has been achieved in our understanding of cellular and molecular phenotypes in the pathogenesis of SLE. Here, we have reviewed the important features of aberrant signaling pathways in SLE T cells. T cells have a vital role in the immune response, whereas other immune cells such as B cells, dendritic cells, macrophages, and neutrophils cannot be ignored in the development of autoimmune diseases. Abnormal activation of the TCR and PI3K-Akt-mTOR signaling pathways and various molecules including PP2A, CaMKIV, CD44, ROCK, mTOR, and SRSF1 affect the function and the differentiation of T cells. Moreover, aberrant cytokine production and the activation of JAK–STAT pathways are also involved in the differentiation of pathogenic effector T cells and impaired Treg cells. In addition to the aberrant pathways described above, alterations in metabolism of immune cells have been recently recognized in patients with autoimmune diseases (113, 117).

Clinical manifestations including symptoms, severities, and clinical response are extremely variable in SLE patients, indicating that no single mediator or pathway can account for the complex pathogenesis. For example, decreased expression levels of CD3ζ are found in many but not all SLE patients (240). The more we understand and elucidate cellular and molecular aberrations in SLE, the more we realize the complexities of the pathogenesis of SLE. However, each aberration has the possibility to be a promising therapeutic target (Table 1). In addition, the analysis of various molecular phenotypes may contribute to patient stratification leading the development of more personalized strategies in SLE treatment.

**Table 1:** Signaling molecules as potential therapeutic targets for systemic lupus erythematosus (SLE).

Molecule	SLE patients	Mice	Targeting studies *in vitro*/*ex vivo*	Pre-clinical	Clinical
CD3ζ	Decreased	CD3ζ ko mice develop multi-organ inflammatory disease	Overexpression in SLE T cells restores Ca^2+^ flux and p-Tyr and IL-2 production		

Calcium/calmodulin kinase IV (CaMKIV)	Activated	Higher activity in T cells from MRL/lpr mice	Inhibition in SLE T cells decreases IL-17 production	Genetic depletion and inhibition with KN-93 are effective in MRL/lpr mice	

Spleen tyrosine kinase (Syk)	Increased	Syk is expressed in the skin lesion of MRL/lpr mice	Inhibition with R406 in SLE T cells	Syk inhibitor is effective in MRL/lpr, New Zealand black/white, and BAK/BAX mice	

Ezrin/radixin/moesin (ERM)	Increased phosphorylation	Moesin-deficient mice develop autoimmune phenotypes	Forced expression of active ezrin enhanced the adhesion and migration in T cells		

Rho associated protein kinase (ROCK)	Higher activity in peripheral blood mononuclear cells from SLE patients	Higher activity in T cells from MRL/lpr mice	Inhibition with ROCK inhibitor in SLE T cells	ROCK inhibitor reduces autoantibodies and proinflammatory cytokine production in MRL/lpr mice	

Calcineurin-nuclear factor of activated T cells (NFAT)	Increased nuclear recruitment/activation of NFATc2	Elevated NFATc1 in MRL/lpr mice			Calcineurin inhibitors widely used

Phosphoinositide-3 kinase (PI3K)	PI3Kp110δ is activated	Activated in T cells from MRL/lpr mice	PI3Kδ inhibitor restores activation-induced cell death in SLE T cells	p110δ inhibitor is effective in MRL/lpr mice	

Mechanistic target of rapamycin (mTOR)	mTORC1 activity is increased, and mTORC2 is decreased	mTORC1 is activated in the livers of MRL/lpr mice		Rapamycin is effective in MRL/lpr mice	Rapamycin is effective, and clinical trial is ongoing

## Author Contributions

TK, VM, and GT conceptualized the article, reviewed the literature, and wrote the manuscript.

## Conflict of Interest Statement

The authors declare that the research was conducted in the absence of any commercial or financial relationships that could be construed as a potential conflict of interest.
